# Transcranial random noise stimulation combined with cognitive training for treating ADHD: a randomized, sham-controlled clinical trial

**DOI:** 10.1038/s41398-023-02547-7

**Published:** 2023-08-02

**Authors:** Ornella Dakwar-Kawar, Noam Mairon, Shachar Hochman, Itai Berger, Roi Cohen Kadosh, Mor Nahum

**Affiliations:** 1 School of Occupational Therapy, Hebrew University of Jerusalem, Jerusalem, Israel; 2 School of Psychology, University of Surrey, Guildford, UK; 3 Pediatric Neurology Unit, Assuta-Ashdod University Hospital and Faculty of Health Sciences, Ben-Gurion University of the Negev, Beer-Sheva, Israel; 4 School of Social Work and Social Welfare, Hebrew University of Jerusalem, Jerusalem, Israel

**Keywords:** ADHD, Human behaviour, Learning and memory

## Abstract

Non-invasive brain stimulation has been suggested as a potential treatment for improving symptomology and cognitive deficits in Attention-Deficit/Hyperactivity Disorder (ADHD), the most common childhood neurodevelopmental disorder. Here, we examined whether a novel form of stimulation, high-frequency transcranial random noise stimulation (tRNS), applied with cognitive training (CT), may impact symptoms and neural oscillations in children with ADHD. We conducted a randomized, double-blind, sham-controlled trial in 23 unmedicated children with ADHD, who received either tRNS over the right inferior frontal gyrus (rIFG) and left dorsolateral prefrontal cortex (lDLPFC) or sham stimulation for 2 weeks, combined with CT. tRNS + CT yielded significant clinical improvements (reduced parent-reported ADHD rating-scale scores) following treatment, compared to the control intervention. These improvements did not change significantly at a 3-week follow-up. Moreover, resting state (RS)-EEG periodic beta bandwidth of the extracted peaks was reduced in the experimental compared to control group immediately following treatment, with further reduction at follow-up. A lower aperiodic exponent, which reflects a higher cortical excitation/inhibition (E/I) balance and has been related to cognitive improvement, was seen in the experimental compared to control group. This replicates previous tRNS findings in adults without ADHD but was significant only when using a directional hypothesis. The experimental group further exhibited longer sleep onset latencies and more wake-up times following treatment compared to the control group. No significant group differences were seen in executive functions, nor in reported adverse events. We conclude that tRNS + CT has a lasting clinical effect on ADHD symptoms and on beta activity. These results provide a preliminary direction towards a novel intervention in pediatric ADHD.

## Introduction

Attention-deficit/hyperactivity disorder (ADHD) is a neurodevelopmental disorder characterized by inattention, hyperactivity, and impulsivity [1], with an estimated prevalence of 5.2% in children worldwide [2]. Deficits in executive functions (EFs), particularly in inhibition, working memory and in sustained attention, have been shown to be key, and potentially a causal, feature of the disorder [3–7]. These are accompanied by under-activation in cortical areas associated with EF, including the dorsolateral prefrontal cortex (DLPFC), the anterior cingulate cortex (ACC), and the right inferior frontal gyrus (rIFG) [8, 9]. In addition, sleep disturbances (e.g., night awakenings, sleep onset difficulties) have also been documented in ADHD [10, 11]. Both EF and sleep disturbances are related to functional impairments in academic, family, and social domains, and may be associated with increased risk for co-morbidities with other psychiatric disorders [12–14].

Current gold-standard treatments for ADHD symptoms include pharmacological treatments, psychosocial interventions, or their combination [15, 16]. Despite their proven efficacy, they are associated with a range of side effects and relatively poor adherence [17], and have potentially limited long-lasting effects [15, 18, 19]. There is therefore a pressing need for developing and testing novel, non-pharmacological interventions for ADHD.

Transcranial electrical stimulation (tES) has been suggested as a possible intervention avenue for children and adults with ADHD [20]. In tES, a weak electrical current is applied to the brain via skin-electrode interface, creating an electric field that modulates neuronal activity. Its excellent safety profile and minimal side effects—which mainly include local itching and tingling during stimulation—make it particularly suitable for pediatric populations [20–23]. Transcranial direct current stimulation (tDCS) is the most studied type of tES in ADHD but the evidence regarding its efficacy in pediatric ADHD is still mixed [24–32].

Transcranial Random Noise Stimulation (tRNS) is a more novel form of tES, in which stimulation is delivered via both electrodes, and which presumably amplifies neural responses via the mechanism of stochastic resonance [33]. Compared to tDCS, which uses one excitatory and one inhibitory electrode, tRNS uses two excitatory electrodes, making it less sensitive to cortical folding, thereby reducing the potential impact of anatomical variations between participants [34]. tRNS was shown to successfully improve cognitive functions in adults [35], and the outcomes of numerical training and mathematics performance in a small sample of children with dyscalculia [36]. We have recently shown that tRNS over the DLPFC and IFC applied concurrently with cognitive training (CT) is favorable to tDCS and CT in improving ADHD symptoms and EFs in a pediatric sample of unmedicated 6–12-year-old children with ADHD; with effects lasting for at least 1 week after treatment completion [37, 38]. However, due to the lack of sham treatment, it was unclear whether the beneficial results for tRNS were due to worsening following the tDCS treatment (see [31]).

The effect of CT alone on clinical symptoms in ADHD is still inconclusive. While one meta-analysis concluded that CT alone has a limited efficacy and transfer effects in ADHD [39], others suggested that it can be an effective intervention for pediatric ADHD ([40], see also [41]). Moreover, a new videogame targeting EF (EndeavorRX^TM^) has recently received FDA clearance as a second-line treatment for ADHD, based on data showing improvements in performance on a sustained-attention test [42]. It has been suggested that the effect of stimulation can be boosted by applying it in combination with CT which improves the specific cognitive function [43], inducing greater plasticity [44] which leads to larger and lasting effects, which can last from 8 days to 6 months [45].

Here, we conducted the first sham-controlled RCT to examine the potential effects of tRNS combined with CT on symptoms, EFs, processing speed (PS), and sleep metrics in unmedicated children with ADHD. Specifically, ADHD has been associated with sleep disturbances [10, 46], but the effects of tRNS on sleep-related metrics have not yet been explored. We further examined, for the first time in ADHD, the effects of stimulation on resting-state (RS) neural activity. Atypical RS-EEG oscillations have been documented in pediatric ADHD (e.g., [27, 47, 48]), potentially indicating atypical cortical activity [49–52]. It has been suggested that tRNS may improve the capacity for sustained attention in individuals with suboptimal cortical arousal, as indexed by reduction in theta/beta ratio [35]. It may also lead to alterations in the amplitude of neural markers, such as the early negative deflection of N1, which is related to attention and preparatory activity and to greater allocation of attentional resources [53]. However, previous studies found no effects of tRNS on modulating RS-EEG activity in participants without ADHD [54, 55].

More recently, studies suggested that electrophysiological signals should be analyzed for both their periodic and aperiodic (1/f-like) properties of the neural power spectra [56]. Specifically, standard analytic approaches of RS-EEG periodic parameters (center frequency, power, bandwidth) could be confounded by other aperiodic features of the power spectrum (i.e., offset, exponent) [57], compromising physiological interpretations. This aperiodic activity reflects the pattern of power across frequencies and is thought to underlie synaptic currents [58]. Using aperiodic analysis, a steeper aperiodic exponent, presumably reflecting atypical excitation/inhibition (E/I) balance in cortical circuits, was found in unmedicated children with ADHD [57]. In contrast, another study on adolescents with ADHD found a smaller “flattened” aperiodic exponent relative to a non-ADHD comparison sample [59]. While this shift could be attributed to various factors, it highlights an imbalance in E/I in cortical circuits which is related to disrupted information processing [60]. E/I has been proposed as a neural marker for stimulation efficacy [61, 62], and could reliably predict ADHD likelihood in early development [59]. The only study to date which examined the effect of tRNS on aperiodic activity found that tRNS increases E/I and is associated with cognitive improvement in adults without ADHD [62]. Here, we examine these effects in unmedicated children with ADHD.

We hypothesized that tRNS combined with CT will lead to improvements in ADHD symptoms (reduction in ADHD-RS scores) and in behavioral and parent-reported EFs (improvement in parent-reported EF scores and in WM and PS behavioral metrics), compared with sham stimulation + CT; and that these effects would endure at follow-up. Finally, based on studies showing modulations in RS-EEG activity following tRNS, we hypothesized that the intervention would lead to modulation in both the periodic and aperiodic neural activity, showing lower aperiodic exponent.

## Methods

### Study design

We conducted a randomized, sham-controlled, double-blind trial of unmedicated children diagnosed with ADHD. The study CONSORT diagram is given in Figure S1. Twenty-five children were assessed for eligibility, 24 were randomized, and 23 participants completed the study. Only 1 participant was excluded from the study, due to difficulties complying with the required frequent arrival to the lab for treatment during the COVID-19 pandemic.

**Fig. 1:**
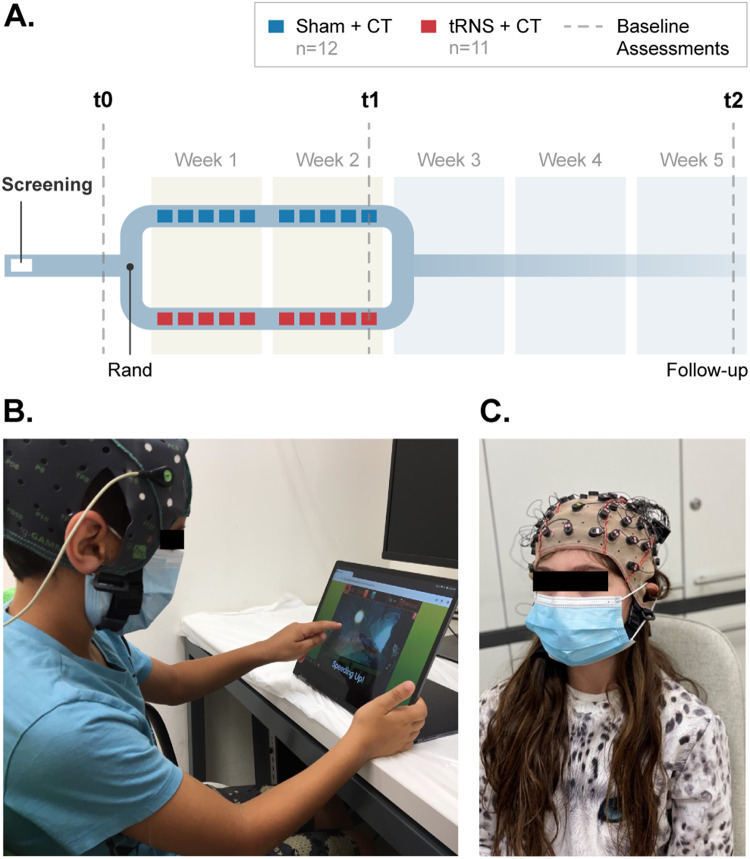
Study design. **A** Following screening, eligible participants underwent baseline assessments (t0) and were then randomized into one of two groups (tRNS + CT or sham + CT). Participants received 10 daily treatment sessions over 2 weeks (1 and 2). Assessments (dashed lines) were repeated at the end of week 2 (t1) and again at follow-up (t2). **B** An example of a tES + CT session. Children sat in front of the tablet which delivers the CT, while receiving tES (active or sham) for 20 min each session. **C** An example of RS-EEG recording session, in which EEG is recorded from children at rest. Pictures of children are included with written permission from participants and their parents. CT cognitive training. Rand randomization.

Study design is depicted in Fig. 1. All children were newly diagnosed and drug naïve. Following screening, eligible participants were assessed at baseline and then randomized into receiving either tRNS + CT (*n* = 11) or sham + CT (*n* = 12) for 2 weeks (weeks 1–2). Each group received their designated treatment for 5 consecutive days each week (one session each weekday). Participants were then assessed again with the same battery at the end of week 2 (t1), and 3 weeks later (t2), to examine endurance of effects. Each assessment session lasted for roughly 3 h. Parents and children, as well as study RAs, were blinded to treatment assignment. The total duration of subject participation in the study was 6 weeks. All study-related activities were conducted in a research lab at the Hebrew University of Jerusalem.

### Study population

Recruitment period was between December 2019 and December 2021. Participants (6–12 y/o) were recruited among children who were referred to the ADHD clinic by their pediatricians, general practitioners, teachers, psychologists, or parents. All participants gave verbal assent for participation and their parents provided written informed consent. All study procedures comply with the ethical standards of the relevant national and institutional committees on human experimentation and with the Helsinki Declaration of 1975, as revised in 2008. All procedures involving human patients were approved by the Helsinki Committee (IRB) of the Hebrew University and Hadassah Medical Center (Jerusalem, Israel). The study is registered at ClinicalTrials.gov (identifier NCT03104972) and was concluded according to the pre-specified protocol with procedural changes in the trial implementation (see further details in Supplementary Material 1).

A power analysis using G-Power [63] showed that the *n* = 20 would allow to detect an effect with a direction hypothesis, given our previous results [37], with power = 0.8, and *α* = 0.05 and an effect size of Cohen’s d of 1.19.

The following inclusion criteria were applied: (1) age between 6–12 y/o; (2) score above the standard clinical cut-off score for ADHD symptoms on the ADHD DSM-5 scales; (3) meeting criteria for ADHD according to DSM-5, using the “gold standard” procedure as described by the American Academy of Pediatrics, which includes a semi-structured interview of the patient and parents by a specialist in pediatric neurology and child development, a neurological examination. Children were excluded from the study if they had one of the following: (1) a chronic neurological disease, epilepsy in the participant or in a first-degree relative, intellectual disability, other chronic conditions, chronic use of medications, or other primary psychiatric diagnosis (e.g., depression, anxiety, psychosis); (2) any Axis-1 disorders, assessed using the Kiddie-SADS-Lifetime Version, Hebrew version, which uses the DSM–5 criteria; (3) girls who began the age of puberty, based on a self- and part-report puberty questionnaire. The tool was translated to Hebrew by the study staff; (4) existence of epileptiform activity based on prospective resting-state electroencephalography performed at screening. EEG records were standardized and recorded with g.Recorder software (gTec, Schiedlberg, Austria), using a 64-channel wireless electroencephalography cap system (g.Nautilus) with gel-based electrodes.

### Outcome measures

#### Primary outcome

The primary outcome measure was ADHD symptom severity, determined using the total score of the parent-reported ADHD-RS diagnostic questionnaire [1]. This scale is of well-accepted validity and reliability, regarded as standards in ADHD diagnosis and treatment effect. The scale contains 18 items based on the wording used to describe those items in the DSM-5: the first 9 items measure inattention (IN) symptoms, while the followed 9 items measure hyperactive-impulsive (HI) symptoms (see full description in [37]).

#### Secondary outcomes

Global functioning was measured using the CGI-S (Clinical Global Impression–Severity) scale [64], memory performance was measured using the Digit Span test [65] and PS was measured using the MOXO-CPT task (NeuroTech Solutions Ltd); These measures have been detailed in our previous publications (see [37, 38]). Everyday EFs were assessed using the Behavior Rating Inventory of Executive Function (BRIEF, [66]), parent and teacher reports.

RS-EEG. The full details on the EEG recording and pre-processing are given in Supplementary Material 1. In short, electrophysiological data was recorded using an eyes open (EO) resting condition in a quiet room for 5 min. Data was acquired using the g.Recorder system (v4.3, hereafter referred to as the research EEG system, g.Tec, Schiedlberg, Austria) connected to a g.Nautilus wireless EEG electrode cap placed on the participant’s head according to the International 10–20 system (Easy Cap), using known anatomical landmarks. We used the standard 32 EEG electrode placements recordings.

Pre-processing of EEG data. EEG data was analysed using EEGLAB software [67], an open-source MATLAB toolbox (freely available from http://www.sccn.ucsd.edu/eeglab/) and custom MATLAB scripts.

A fast Fourier transform (FFT) was used to calculate the absolute power spectra within different specific frequency bands, focusing on delta (0.5–2 Hz), theta (34–7 Hz), alpha (8–13 Hz), beta (13–30 Hz), and total power (1–40 Hz) of all band changes in each group. Here, we focused on analysing the data from electrodes over the stimulation sites (F3, F8) as well as from frontal midline area (Fz), which has been shown changes in aperiodic exponent following tRNS applied to similar brain regions [62]. FFT has been extracted for each electrode.

We then employed a spectral parameterization approach which enables decomposition of the neural signal into its respective periodic and aperiodic components, with a tool called FOOOF (fitting oscillations and one over *f*) [56]. Importantly, the FOOOF tool calculates both the aperiodic value for each electrode and models the distribution features of the periodic component in the bands of interest. This also gives the central frequency and bandwidth of the periodic component’s distribution. We note that we were not able to detect the peak for alpha and theta frequencies for most participants, and therefore did not have enough data to draw reliable conclusion for these frequencies. We therefore do not report results for these frequencies.

#### Exploratory outcomes

Sleep quality was assessed using the Hebrew version of the Pittsburgh Sleep Quality Index [68], a self-report questionnaire used to assess sleep quality and disturbances, designed for adults (see Supplementary Material 1). Here, children were asked to answer this questionnaire together with their parents, and one item was adapted to fit children’s daily life (the item ‘use of sleeping medication’ was replaced by ‘how many times you wake up at a night’).

### Study interventions

A detailed description of the study interventions is given elsewhere ([37, 38]). In short, participants completed computerized CT along with either tRNS (tRNS + CT arm) or sham (sham + CT arm) for 20 min/day for 10 days during a 2-week period. Sessions were conducted daily each week from Sunday through Thursday, and no sessions were conducted on the weekend (Friday and Saturday). The intervention times were set individually based on the personal preferences and availability of each participant and were kept fixed throughout the intervention period.

For sham tRNS we used the same montage as in active tRNS, but here the 30 s of ramp up of the current from 0 to 0.75 mA was immediately followed by 30 s ramp down period to 0 mA, such that participants did not receive active stimulation between ramp-up and down. This method has been shown to provide effective blindness of the stimulation condition as both active and sham tES would lead to slight itching sensation that would disappear due to scalp habitation [69].

### Randomization and blinding

Participants were randomized in a 1:1 allocation ratio to receive active or sham tRNS. Three staff members with no contact with participants and who were not involved in any other study procedures or data analysis generated balanced random samples throughout the course of an experiment, using Smith’s randomization algorithm based on the variance minimization procedure [70], and programmed the device to discharge sham/active stimulation according to each participant’s allocation. Our previous research [70] highlighted the advantages of variance minimization over prevalent random assignment procedure in terms of reducing the Type I error rate and providing accurate estimates of the effect of the group on the outcome variable. The active and sham tRNS were physically indistinguishable based on the electrode locations and the displayed information for RAs and participants. RAs who were involved in administering treatment sessions were not involved in data analysis. Participants and their parents were blind to the treatment assignment, as well as the PIs and study staff involved in training and/or assessments or data analysis. To examine the success of the blinding procedure, parents of participating children were asked at the end of the experiment which intervention they think that their child received and to rate the level of confidence in their prediction. The blinding assessment was performed using the Bang Blinding Index, ranging from −1 to 1, with 1 indicating total lack of blinding, 0 indicating complete blinding and −1 indicating opposite guessing which may be related unblinding [71]. A positive value suggests that parents correctly guessed their child’s treatment allocation beyond chance.

### Statistical analysis

All statistical analyses were conducted using R. Study staff who conducted the analyses were blind to group assignment during pre-processing and analysis of all measures. Overall, there was less than 4% missing data in the entire dataset, which stem from missing data in the scales of BRIEF teachers and RS-EEG recordings, as well as missing daily treatment sessions. This was due to movement restrictions imposed during the COVID-19 pandemic, which affected arrivals to the lab and to schools. Before statistical testing, outlier data, defined as values 2.5 SDs above or below the group mean of each measure, were removed from further analyses [72]. The range of outliers across variables did not exceed 3% in the behavioral outcomes and were solely in the PS and sleep index scales, while in the RS-EEG recordings it did not exceed 6% and was just in F3 electrode. There were no significant group differences in terms of missing data, nor in outlier variables in all time points (*p* > 0.5).

Demographic characteristics of age, gender, and estimated IQ (calculated based on the Vocabulary and Block Design dyad subscales of WISC as short-form IQ assessment [73]) were compared using Student’s t-tests and chi-square tests for independent samples. ADHD symptoms were compared between groups using separate one-way MANOVAs, using the IBM SPSS Statistics version 25 (IBM Corp., Armonk, N.Y, USA). Linear mixed effects models (LMMs) were used to examine treatment effects. LMMs account for within-subject correlations and for associations induced by repeated measurements. To conduct LMM analyses, we used the R-package *nlme* with maximized log-likelihood on the outcome measures, and subjects as the random factor. We examined outcomes immediately post-treatment (t1) and at a 3-week follow-up (t2) for each condition and included treatment arm (tRNS + CT, Sham + CT) and time (t1 and t2) as predictors. Baseline performance was added as a covariate to the model, allowing for better adjustment for minor differences in the pre-treatment means.

For our primary outcome measure (ADHD-RS), a simple model which included the main effects of stimulation and time with no interaction between them was preferred to a more complex model that included the interaction term (F(7) = 2.06, *p* = 0.15; see also [37]). This is further justified given that the group X time interaction was not significant (F(21) = 1.38, *p* = 0.18). We therefore report this parsimonious model for the secondary measures as well. For all measures, we verified that the residuals were normally distributed using a q-q plot and the Shapiro-Wilk normality test. The only exceptions were the SOL index residuals, and some of RS bands (rIFG: delta, alpha and beta; lDLPFC: delta and alpha) which were not normally distributed; we therefore applied log10 transformations to normalize these measures.

## Results

### Demographic characteristics of the study population

Demographic characteristics of the sample are given in Supplementary Table S1. There were no significant differences in age, estimated IQ, or symptom severity between the two groups.

### Side effects and safety issues

We assessed adverse events that were spontaneously reported during the treatment sessions and at the end of each session. In this survey we included open-ended questioning rather than asking about specific side effects, as this has been shown to yield more credible reports in this age group (see [74]).

The full list of side effects is given in Supplementary Table S2. Overall, there were 117 records of side effects reported during the trial, and none of them were considered clinically significant. The most common side effects were itching (27% and 33% of sessions in the active and sham groups, respectively), followed by discomfort (6% of sessions) and difficulty concentrating (5% of sessions). There were no significant between-group differences on all reported side effects (*p* > 0.07).

### Primary outcome measure

Compliance with treatment was high and did not significantly differ between groups (9.91 ± 0.29 vs. 9.83 ± 0.55 for mean ± SD sessions for active vs. sham groups, respectively; F(21) = 0.75; *p* = 0.39).

**Fig. 2:**
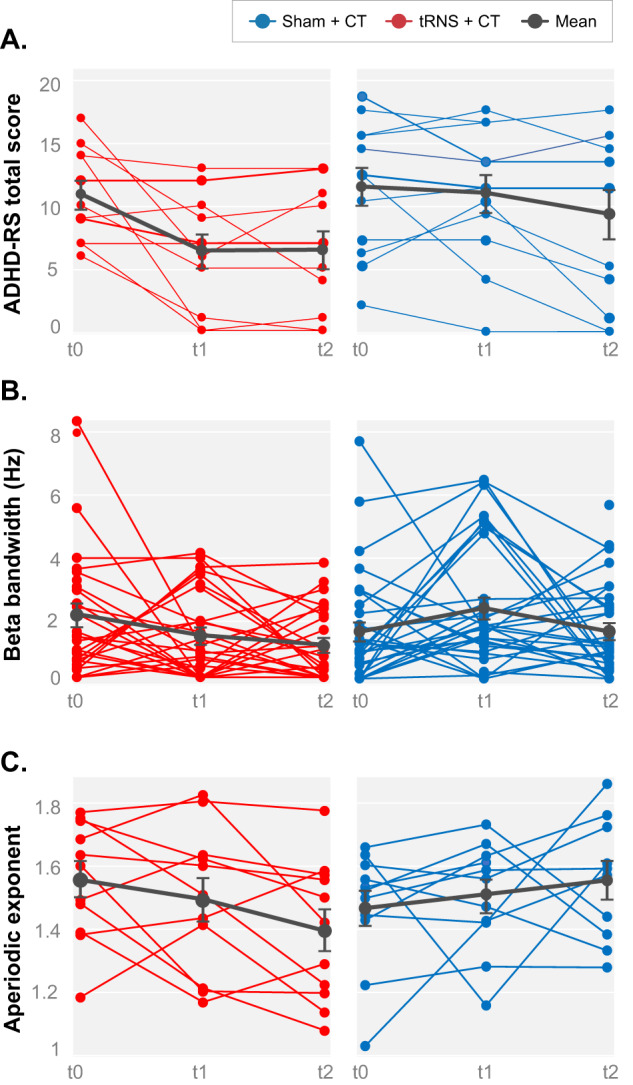
Clinical symptoms and RS-EEG periodic and aperiodic measures following treatment. Graphs show scores for both active (left, red plots) and sham (right, blue plots) group participants for the 3 time points (t0–t2). **A** ADHD-RS total scores (higher scores indicate more severe clinical symptoms). **B** Periodic parameter of the beta bandwidth (Hz). **C** Aperiodic exponent parameter. Lines represent individual participants’ scores. Black lines represent mean scores ± SEM across each group.

The results of the analyses of the primary outcome (ADHD-RS total score) are given in Table 1 and individual data is shown in Fig. 2A. Following treatment, the mean ADHD-RS total score was 6.36 ± 1.37 and 10.58 ± 1.46 for the active vs. sham groups, respectively. There was a main effect of stimulation, indicating decreased ADHD symptoms following tRNS + CT compared to sham + CT. Moreover, there was a non-significant effect of further decrease in symptom severity from post-treatment to follow-up. The estimated effect size of the treatment predictor was Cohen’s d of -0.94.

**Table 1:** A regression model of primary and secondary outcome measures post-treatment (t1) and at a 3-week follow-up (t2), while covarying for baseline scores.

	Beta weights (standardized)	Std error	DF	*t* value	*P* value
*Clinical symptoms (ADHD-RS)*					
Intercept	−0.34	0.27	22	1.23	0.0078**
Baseline score	0.66	0.15	20	4.53	0.0002***
Treatment	−0.61	0.29	20	−2.11	0.047*
Time	−0.15	0.12	22	−1.25	0.22
*Sleep Index total score*					
Intercept	−0.44	0.413	20	−1.07	0.3
Baseline score	0.12	0.06	19	1.87	0.08
Treatment	0.48	0.24	19	2	0.059
Time	−0.14	0.23	20	−0.62	0.54
*Sleep Onset Latency score*					
Intercept	−0.67	0.38	22	−1.78	0.9
Baseline score	0.31	0.16	20	1.92	0.07
Treatment	0.66	0.28	20	2.37	0.02*
Time	0.11	0.21	22	0.56	0.58
*Wake-Up score*					
Intercept	−0.33	0.4	22	−0.82	0.42
Baseline score	0.23	0.17	20	1.36	0.19
Treatment	0.6	0.28	20	2.13	0.046*
Time	−0.12	0.22	22	−0.56	0.58
*RS-EEG measures periodic Beta bandwidth*					
Intercept	0.75	0.28	56	2.72	0.009**
Baseline	0.06	0.09	56	0.69	0.5
Treatment	−0.55	0.18	56	−3.03	0.004**
Time	−0.33	0.16	56	−2.05	0.04*
*Aperiodic exponent (Fz)*					
Intercept	0.71	0.26	21	2.74	0.01*
Baseline	0.58	0.17	20	3.41	0.002**
Treatment	−0.11	0.06	20	−1.99	0.06
Time	−0.02	0.04	21	−0.6	0.55

*Std* standard, *DF* degrees of freedom.

*p < 0.05; **p < 0.005; ***p < 0.0005.

Bold values indicate significant differences at a level of *p* < .05.

Based on an acceptable cutoff for treatment response (30% ADHD-RS symptom reduction; see [75, 76]), we found that 6/11 participants (55%) in the tRNS + CT group achieved clinically meaningful treatment response post-treatment, compared to 2/12 participants (17%) in the sham group. At follow-up, 7/11 participants (64%) in the active group reached clinically meaningful response, compared to 4/12 participants (33%) in the sham group.

### Secondary outcome measures

The secondary outcome measures were considered more exploratory and hence we present them below without applying a correction for multiple comparisons. Still, we note that none of the secondary measures were significant at a *α* ≤ 0.05 after applying FDR correction for multiple comparisons.

#### Clinical symptoms (CGI-S)

There was no significant post-treatment effect of stimulation type (see Figure S2A and Table S3). However, there was a significant effect of time, indicating reductions in clinical symptoms in both groups at follow-up compared to post-treatment.

#### Cognitive outcomes: WM, STM, EF, and PS

There were no statistically significant effects of treatment type nor of time on any of the EF or PS measures used (see Supplementary Material, Figure S2, Table S3).

#### Sleep-related metrics

The results of the LMMs predicting sleep-related metrics are given in Table 1. For the total sleep index score, there was a marginal effect for stimulation type post-treatment (β = 0.48 (SE = 0.24), t(19) = 2, *p* = 0.059), but no significant effect of time, indicating marginal worsening in parent-reported sleep quality following active vs. sham. For the SOL subscale, there was a significant effect of treatment, with no significant effect of time, indicating longer latencies to falling asleep following active treatment, with no significant changes at follow-up. Similarly, for the wake-up times subscale (i.e., the number of times the child woke up during the night), there was a significant effect of treatment, with no significant effect of time, indicating worse wake-up scores following active treatment, with no significant changes at follow-up. There were no significant effects on any other components of the sleep index.

#### Periodic RS-EEG activity

RS-EEG results are summarized in Table 1. To examine the effects of treatment on frontal area, we added to the model the electrode as a random intercept which included data from stimulated sites (F3 and F8), in addition to data from midline frontal electrode (Fz).

There was a main effect of stimulation in the beta bandwidth of the extracted peak, indicating narrower beta bandwidth post-treatment following active compared to sham stimulation. There was a significant effect of time, indicating further reduction in beta bandwidth at follow-up compared to post-treatment (Fig. 2B). No significant effects were seen on other peak parameters such as power of beta, theta, or alpha peak (see Supplementary Table S3).

#### Aperiodic RS-EEG activity

When using a directional hypothesis, based on the only other tRNS study that examined its effect on aperiodic exponent, significant changes in the aperiodic activity in Fz were seen following active compared to sham tRNS (β = −0.11 (SE = 0.06), t(20) =−1.99, *p* = 0.03), indicating lower aperiodic exponent (i.e., higher E/I). However, we recognize that such results would be considered as non-significant (*p* = 0.06) when using a non-directional hypothesis (see Discussion). There was no significant effect of time, indicating that effects did not significantly change at follow-up (see Table 1, Fig. 2C).

**Fig. 3:**
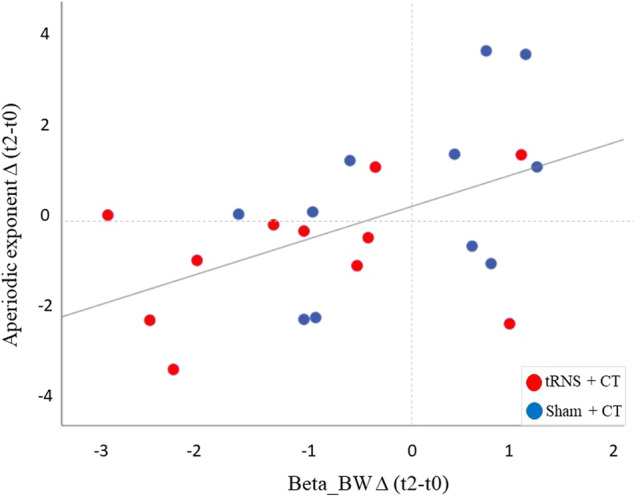
Pearson correlation between periodic (beta bandwidth) and aperiodic exponent changes. The correlation was significant (*r* = 0.503, *p* = 0.013) across the entire sample, indicating that higher E/I (reduced aperiodic exponent) was associated with reduced beta bandwidth from t0 to t2.

Finally, there was a significant correlation between change in activity from t2 to t0 in the periodic beta bandwidth averaged over the frontal area and the aperiodic exponent measure across the entire sample (*r* = 0.503, *p* = 0.013; see Fig. 3).

### Blinding integrity

Parents answered the questions related to blinding, as children showed difficulty in understanding the question. Table S4 lists the parents’ guesses based on blinding assessment. In the active group, the Bang Blinding Index was 0.09 (95% CI, −1.46 to 1.64), suggesting 9% of correct guesses. For the sham group, the Bang Blinding Index was −0.5 (95% CI, −2.08 to 1.08), indicating a pattern of random distribution of responses.

Next, due to the effect of subjective beliefs about receiving an intervention [77], we analyzed the potential differences in blinding success across both groups using a *χ*^2^ test of independence. There were no significant associations between active stimulation guess rate and subjects’ group assignment, *χ*^2^(1, *N* = 16)= 0.78, *p* = 0.38, indicating that the feeling of receiving active stimulation did not depend on the treatment group (Table S4). Moreover, replacing the group predictor (active/sham) with subjective stimulation did not reveal any effect of subjective stimulation (Table S5), confirming that the current effects are due to the actual stimulation given rather than due to a placebo effect.

## Discussion

We conducted a randomized, sham-controlled, double-blind clinical trial to examine the effects of active tRNS + CT in 23 unmedicated children with ADHD. We found that tRNS + CT improved clinical ADHD symptoms immediately following treatment, and that the effects did not significantly change at a 3-week follow-up, compared to the control group of sham + CT. This improvement was accompanied by changes in periodic RS-EEG activity, of reduced bandwidth of extracted peaks in beta in frontal area following active treatment, with further reduction at a 3-week follow-up. Moreover, a trend for lower aperiodic exponent was seen in Fz following treatment. Of the remaining secondary outcomes, the only significant effect was found for longer onset sleep latencies and more wake-up times following treatment compared to control intervention. Finally, adverse effects were minimal and similar across groups.

### Changes in clinical symptoms of ADHD following tRNS + CT

The most notable result is the improvement in parent-reported ADHD symptoms following treatment. These results are in line with our pilot study [37], showing better outcomes—namely improvements in clinical symptoms, WM and PS—for tRNS + CT compared with tDCS + CT. No other study, to our knowledge, has examined the effects of tRNS + CT (or of tRNS in general) in pediatric ADHD. Still, our results are also in accordance with those of previous studies involving tRNS + CT in other pediatric populations [36] as well as in young adults performing cognitive training tasks [35, 53, 78–80]. In contrast, studies involving tDCS + CT in pediatric ADHD samples have not shown consistent results in reducing ADHD symptoms, with the largest RCT in pediatric ADHD to date showed null effects of tDCS on clinical symptoms [31]. Several recent meta-analyses concluded that there is limited evidence of improvements in clinical symptoms [27, 29, 81]. However, these effects are strongly dependent on stimulation parameters [28], and future studies should examine the parameters that could yield beneficial effects using tDCS.

Of note, the effect size found here is comparable to that of pharmacological effective treatment (e.g., SMD of −0.78 for Methylphenidate for a repeated intake over 12 weeks) [17]. Still, the small sample size calls for extension and replication of these results in larger, fully powered studies.

The clinical improvement in attention symptoms following tRNS + CT may be accounted for by improvements in cortical attention networks. Attention has been shown to depend on both intra and inter-fronto-parietal-temporal synchronization [78, 82, 83]. Effective communication within this network requires strong-enough signal-to-noise ratio (SNR) [78], yet not all the neurons can reach the appropriate threshold for depolarization, and this characteristic might be more excessive in atypical development and have subsequent effect on neuroplasticity [20]. Stochastic resonance—the presumed mechanism underlying tRNS—was suggested to improve the inter-regional transmission [84] and synchronization [85], by improving the SNR in the attention network [78], potentially by increasing the level of neuronal Excitation-Inhibition ratio [62].

### Effects of tRNS at 3 weeks of follow-up

Our findings further point to a lasting effect of tRNS + CT. We found a non-significant effect of time, indicating non-significant changes in treatment effects at a 3-week follow-up compared to post-treatment. These results are in accordance with our previous pilot studies showing effects lasting for at least 1 week following 5 treatment sessions [37, 38]. Other studies also reported effects lasting from 8 days to 6 months following 3–5 stimulation sessions in adults without ADHD [86–90]. At the neural level, multiple brain mechanisms underlying long-term effects for tRNS have been suggested, from cellular and molecular mechanisms to neuronal and hemodynamic effects [21, 62, 90, 91]. Future studies should examine the mechanisms that underline the effects observed in our study.

### Changes in periodic and aperiodic RS-EEG activity following treatment

We found reduced bandwidth of the extracted peaks of beta in electrodes above the areas in which active tRNS was applied (lDLPFC and rIFG) and in electrode Fz, compared to the control group. A further reduction in beta bandwidth was seen at 3-weeks of follow-up. Note that there was no significant change in beta power, neither in center of frequency. Furthermore, in Fz, a site that has been shown an effect of tRNS in increasing E/I [44], an effect for lower aperiodic exponent was seen following treatment compared to control.

Changes in the bandwidth of frequencies are underreported and have been suggested to reflect firing rate of neural population [92] and neuroplasticity (synaptic pruning) [93]. Changes in the aperiodic exponent have been linked to the E/I of field potentials [60], with lower aperiodic exponent attributed to higher E/I, and neuroplasticity [62]. Moreover, it may reflect increased signal-to-noise ratio, increased GABA or reduced glutamate signaling [60, 94]. Recent evidence suggests that the aperiodic exponent may underlay a range of cognitive and behavioral states [59, 95, 96], and that the E/I balance is related to reaction time variability in adolescents with ADHD [59]. The E/I balance could also be used as a marker associated with ADHD risk, with larger aperiodic exponent associated with greater family history of ADHD in infancy, while in adolescence, ADHD diagnosis was associated with a smaller aperiodic exponent [97]. It has been suggested that tRNS, which is an excitatory form of neurostimulation [33, 34], could increase E/I, as reflected by lower aperiodic exponent [62]. Our findings here are in line with these suggestions which were derived from studies in non-clinical samples of adults [57, 62]. The link between changes in E/I and changes in the beta bandwidth found here, as well as in previous studies (see [56], [92, 98]) further strengthens the notion that delivering electrical random noise to the brain influences the underlying electrophysiological signal, leading to increase in excitation [34]. However, while our findings are in line with previous findings, we recognize that our results are based on a relatively modest sample size and a directional hypothesis in the case of the aperiodic exponent.

Our results further highlight the need to use aperiodic analysis of RS-EEG data. Previous spectral power analyses, which refer to the oscillatory brain activity, have suggested that increased ratio of theta to beta power (TBR) during RS in ADHD population could serve as a neural marker, helping with the diagnosis of this population [99]. However, more recent examinations of the reliability of TBR as a diagnostic marker for ADHD, which calculate it using fixed frequency bands [57], led to conflicting results [51, 100, 101]. Recently, it has been suggested that these discrepancies could be accounted for by confounding effects of other relevant features of the power spectrum, including misestimating spectral power since participants vary in center frequencies [102], in addition to shifts in peak oscillation frequency and altered slope or offset of the aperiodic component of the power spectrum [57]. Analysis of the aperiodic activity in the spectrum, which reflects the pattern of power across frequencies and thought to underlie synaptic currents [58], could therefore be useful when parameterizing the neural power spectra [56]. More research is needed to examine the changes in periodic and aperiodic neural activity following tRNS.

### Changes in sleep-related metrics following treatment

Following treatment, participants reported longer SOLs and more wake-up times. The increase in wake ups during the night are consistent with a few tDCS studies [103–105]. However, other studies failed to find stimulation effects on sleep indices [106, 107]. According to a recent review, anodal tDCS, which is an excitatory form of stimulation similar to tRNS, increases the duration of wake periods without affecting the frequency of awakenings [108]. The potential neural mechanism of tDCS on sleep continuity may be explained by polarity-specific changes in cortical arousal, indexed by RS-EEG gamma band, with reverse effects after cathodal stimulation, extending to subcortical arousal networks via cortico-thalamic feedback loops [103]. One possibility for the worse effects of tRNS on sleep is that random stimulation excited these networks. Unpublished data in 301 adults who received 11 daily tRNS over the bilateral DLPFC along with EF training has shown that reduced hours of sleep leads to better performance on cognitive training (James Sheffield et al., in preparation). Future studies should therefore examine the effects of tRNS on sleep using larger samples and using methods which provide better insights into the different components of sleep.

### Null effects on secondary outcome measures

Analysis of the secondary outcomes showed no effect of treatment on parent/teacher-reported nor on performance-based EFs. Findings in the tRNS literature are mixed regarding the potential effects of stimulation on EFs [35, 86, 109, 110]. Still, the null effect of stimulation on WM and PS is surprising, given our previous results from children with ADHD showing improvements in WM and PS following tRNS + CT compared to tDCS + CT [37, 38], in the same age cohort. However, a few other studies also reported lack of effects on WM in adult [111, 112] samples. The null effect on PS contradicts findings that reported improvements in PS of cognitive tasks following tRNS in adults without ADHD [35, 78, 86, 90, 109].

There are a few potential accounts for the lack of effect on EF-related measures. First, the stimulation duration and site may have been less effective for EF improvement. Changes in EF were seen following pharmacological and psychosocial interventions that lasted between 4–12 weeks [113–118], potentially indicating that longer duration of treatment is needed to drive changes in EF. In relation to stimulation site, while there is no consensus regarding the optimal montage for clinical efficacy in ADHD [25, 81], it is possible that targeting different sites, such as bilateral DLPFC and IFC-parieto-cerebellar networks or even other pre-frontal striatal circuits [8, 36, 90, 119] may yield better outcomes. In addition, the fact that our sample had overall relatively mild symptom severity and mild executive dysfunctions may have contributed to the lack of effects. Finally, since ADHD is associated with late chronotype (i.e., eveningness and delayed sleep onset) and with circadian rhythm disruption [11], this could hinder or abolish tES-induced plasticity and EFs, including WM [120, 121]. Future studies should determine the optimal stimulation parameters (e.g., dose and time of day of stimulation) for driving EF changes and the characteristics of patients (i.e., ADHD subtype, executive dysfunction severity, chronotype) who can potentially benefit from this type of intervention.

### Study limitations and future directions

Despite its strengths, our study has several limitations that should be noted. First, although the sample size was reasonable compared to previous studies in this field [36, 81], it is suitable to detect a large effect size, similar to one that we have observed in a previous study [37], and the effect size we observed for the treatment’s main effect is in line with a meta-analysis which included only 4 tRNS studies [122]. This effect size could be inflated, and the small sample size therefore limits our ability to detect treatment effects with a medium or smaller effect size. Low power also reduced the likelihood that significant results reflect a true effect (see [123]). The small sample size further limited our ability to perform additional analyses of interest, such as analyzing the data for AD/HD subtypes. In addition, although we asked parents regarding treatment assignment at the end of the trial, this was not examined during treatment and the kids were not asked, due to their relatively young age.

Another limitation is that the relatively mild severity profile of symptoms and executive dysfunction in our sample may not be representative of more severe cases. Future studies should include a more heterogenous and a larger sample, with extended stimulation protocols that may be individually tailored to each child’s clinical profile. Such individually-tailored approach should consider several factors, including ADHD subtype and the time of day in which stimulation is given, which should match the child’s chronotypical profile [120]. Recent studies are starting to explore the option of using tES as a remotely monitored home treatment [25]. These options should be further explored the results from our proof-of-concept study in future large, pre-registered studies with well-blinded controls and blinding integrity tests for study staff.

## Conclusions

Our findings have scientific, as well as potentially clinical implications for pediatric ADHD. These findings add to those of our previous investigation [37, 38], and support the efficacy of tRNS + CT in improving ADHD symptoms. The relatively maintained effects of short duration of treatment, along with its excellent safety profile, allow adding its translation to a potential standard-of-care that should be examined further carefully.

### Supplementary information

Supplementary Material 1

Supplementary Material 2
