# Correction: Transcranial random noise stimulation combined with cognitive training for treating ADHD: a randomized, sham-controlled clinical trial

**DOI:** 10.1038/s41398-023-02588-y

**Published:** 2023-08-30

**Authors:** Ornella Dakwar-Kawar, Noam Mairon, Shachar Hochman, Itai Berger, Roi Cohen Kadosh, Mor Nahum

**Affiliations:** 1 School of Occupational Therapy, Hebrew University of Jerusalem, Jerusalem, Israel; 2 School of Psychology, University of Surrey, Guildford, UK; 3 Pediatric Neurology Unit, Assuta-Ashdod University Hospital and Faculty of Health Sciences, Ben-Gurion University of the Negev, Beer-Sheva, Israel; 4 School of Social Work and Social Welfare, Hebrew University of Jerusalem, Jerusalem, Israel

**Keywords:** ADHD, Human behaviour, Learning and memory

Correction to: *Translational Psychiatry* 10.1038/s41398-023-02547-7, published online 02 August 2023

In the original version of this article, the first and last names of all authors were incorrectly constructed. The first and last names were switched. The correct names are: Ornella Dakwar-Kawar

Noam Mairon, Shachar Hochman, Itai Berger, Roi Cohen Kadosh, Mor Nahum.

The original article has been corrected.

