# Supplementary material for: Transcranial random noise stimulation combined with cognitive training for treating ADHD: a randomized, sham-controlled clinical trial
Source: Transl Psychiatry. 2023 Aug 2;13:271. doi: 10.1038/s41398-023-02547-7 (PMC10394047; doi:10.1038/s41398-023-02547-7)
Supplement: Supplementary file 1 — Supplementary Material 1 [file 41398_2023_2547_MOESM1_ESM.docx]

Supplementary Material 1


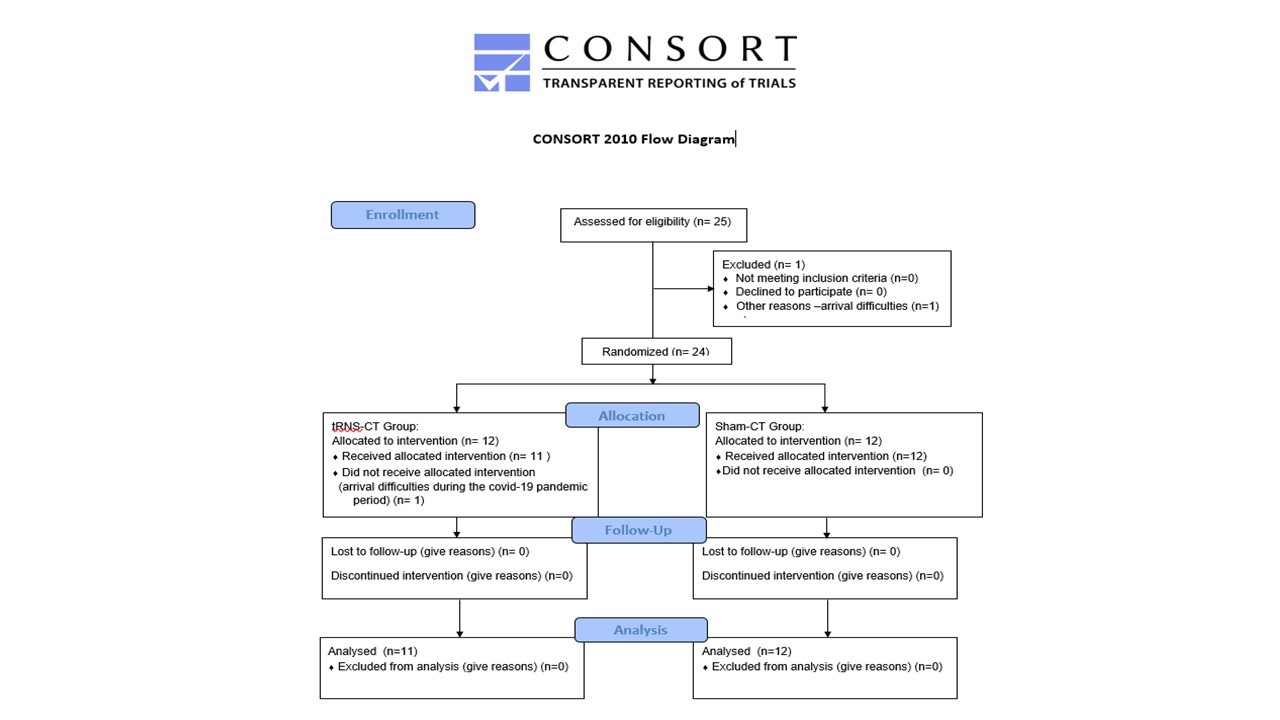


**Figure S1.** Consolidated Standards of Reporting Trials (CONSORT) flow diagram of the progress through the phases of the randomized crossover study of the two groups.

**Additional information on secondary outcomes**

Everyday Executive Functions (EFs) were assessed using the Behaviour Rating Inventory of Executive Function (BRIEF, Gioia et al., 2000), parent and teacher reports. This scale measures the neuropsychological constructs of EF in everyday situations for children and adolescents aged 5-18 years. It consists of 86 items and is completed by a parent and/or a teacher. Items ratings range between 1 (never), 2 (sometimes) and 3 (often). Raw scores are converted to t scores, with t score of 65 or above considered clinically impaired (SD 10). The questionnaire has 8 subscales which correspond to different executive abilities (inhibit, shift, emotional control, initiation, working memory, plan/organize, organization of materials and monitor), two indices (Behavioural Regulation Index and Metacognitive Index) and a Global Executive Composite (GEC), comprised of the sum of scores in all subscales. The Hebrew version of the scale has good psychometric properties for children with ADHD including internal consistency, discriminant validity and test-retest reliability (Gioia et al., 2000; Linder et al., 2010; McCandless & O’Laughlin, 2007). The test-retest reliability of the scale in our sample was good (rs=.67-.85 and .84-.89 for parent and teacher forms, respectively, over a 3-week interval), and the internal consistency was high (α= .93 and α=.834 for parent and teacher forms, respectively).

Processing Speed (PS) was assessed using the MOXO-CPT (NeuroTech Solutions Ltd), a standardized computerized test that measures attentional performance(Berger et al., 2017). The test was performed in a clear examination room in the lab, on a computer screen (36 X 48 cm), which participants were seated at 60cm from the screen. The test consists of 8 levels, each contains 53 trials and lasts for 114.15s, for a total test duration of 15.2 min. On each trial, a stimulus (target or non-target) appears in the middle of the screen for a variable duration of time (0.5, 1, or 3s), followed by a **“**void**”** of the same duration. Of the 53 trials on each level, 33 include target stimuli and 20 contain non-target stimuli. Participants are instructed to respond to the target stimuli by clicking on the spacebar once, and only once, and withhold response to non-target stimuli. PS is derived from the timing index of the MOXO-CPT and is operationalized as the number of correct responses obtained while the target stimulus is still on the screen.

Sleep quality was assessed using the Pittsburgh Sleep Quality Index(PSQI; Buysse et al. 1989), a self-report questionnaire used to assess sleep quality and disturbances, designed for adults. The scale has a validated Hebrew version validated (Oksenberg, 2007). The questionnaire includes 19 individual items which generate 7 “component” scores: subjective sleep quality, SOL, sleep duration, habitual sleep efficiency, sleep disturbances, use of sleeping medication, and daytime dysfunction. The sum of scores for these 7 components yields one global score (range 0-21), with higher scores indicate worse sleep quality. In the current study, children were asked to answer this questionnaire together with their parents, and one item was adapted to fit children’s daily life (the item ‘use of sleeping medication’ was replaced by ‘how many times you wake up at a night’).

RS-EEG

EEG recording. The electrophysiological data was recorded in a quiet room for 5 minutes, while participants were seated on a comfortable chair and were instructed to stay relaxed and still for the duration of the recording. Electrode fitting took ~45 min, during which participants were familiarized with the testing equipment and the procedure. The experimenter sat with the participants in the room during data acquisition, and recordings were paused if the participant showed signs of fatigue or restlessness.

We used the standard 32 EEG electrode placements recorded using the 64-electrode g.Tec cap. Electro-Gel was used as the conducting medium. The right Linked Ear (LE) served as online reference, and the ground electrode was positioned between FPz and Fz, with all electrodes physically referenced to Cz during recording (fixed by the EGI system). The ongoing EEG recording was continuously monitored by another experimenter on a different screen located in the control room, to ensure data quality and to control participant’s vigilance. Online EEG was sampled at 250 Hz and all electrode impedances were kept below 30 kΩ.

Pre-processing of EEG data. EEG data were offline treated with a Hamming windowed sinc Finite Impulse Response (FIR) filters between 0.5-30Hz implemented using EEGLAB’s pop_eegfiltnew function, with no change was made to the sampling rate. Flat channels were removed, and noisy electrodes (large visible drifts or net noise) were interpolated from neighboring channels. All channels were then re-referenced offline to the average of all electrodes. The first 5 seconds were removed from the artifact-free EEG data, and the remaining record was used for data analysis. Artifacts were identified in a semi-automated manner, with sections of data exceeding 200μV being automatically removed. Then, a visual inspection was made to identify and exclude sections of EEG trace containing substantial artifacts. Next, using runica algorithm (pop_runica function), we identified ICA components related to eye and muscle movements by visual inspection, based on their scalp topography, power spectra and time course (Hipp, Siegel, Deouell, & Hebrew, 2013). These eye components were removed from the signal (.43 ± .5 on average). All other components were used for further analysis. Then, all datasets were visually inspected and sections of data containing residual artifacts were removed manually.
