# Supplementary material for: Transcranial random noise stimulation combined with cognitive training for treating ADHD: a randomized, sham-controlled clinical trial
Source: Transl Psychiatry. 2023 Aug 2;13:271. doi: 10.1038/s41398-023-02547-7 (PMC10394047; doi:10.1038/s41398-023-02547-7)
Supplement: Supplementary file 2 — Supplementary Material 2 [file 41398_2023_2547_MOESM2_ESM.docx]

Supplementary Material 2


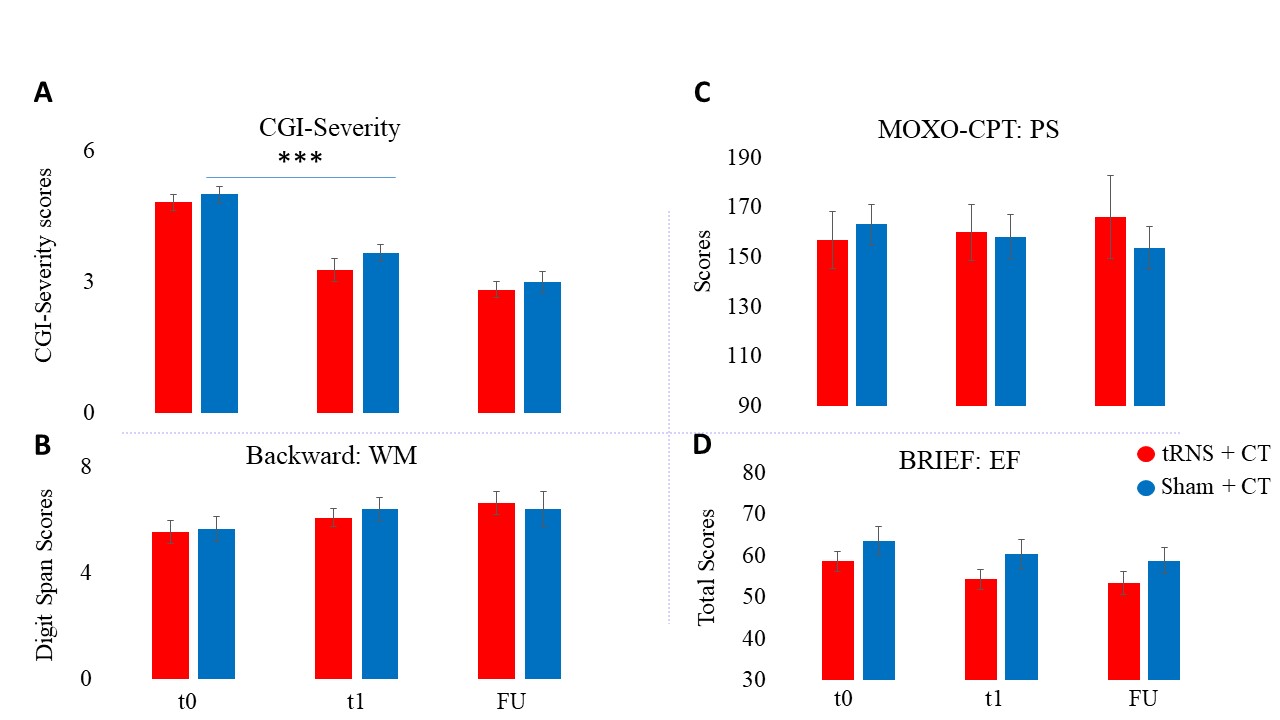


**Figure S2.** Clinical and behavioral symptoms following tRNS+ CT (red bars) vs. sham + CT (blue bars) at baseline (t0), post-treatment (t1) and follow-up (t2). **A**. Clinical symptoms (CGI-S severity). **B.** WM (digit backward span test of WISC). **C.** Processing speed (PS; MOXO-CPT timing index). **D.** Parent-reported EFs (BRIEF-p total score).

**Supplementary Table S1.** Baseline Demographic and Clinical Characteristics

| **Demographic characteristics of the study sample** | | | | | |
| --- | --- | --- | --- | --- | --- |
| **Characteristics** | **tRNS + CT (n=11)**  **mean (SD)** | **Sham + CT (n=12)**  **mean (SD)** | ***t*/χ2/ F**  **(dfh, dfe)** | **P Value** | **Effect Size**  **(partial** η^2^**)** |
| Age (y) | 9.25 (1.42) | 8.64 (1.43) | 1.13 | .3 |  |
| Male (*n*, %) | 10, 90.9% | 10, 83.3% | .29 | .59 |  |
| **WISC** |  |  |  |  |  |
| Estimated IQ | 104.5 (10.76) | 90.8 (21.2) | 2.05 | .067 |  |
| **ADHD symptoms (ADHD-RS)** | Wilk’s Λ = .99 | | .009  (1,21) | .99 | .001 |
| Total score | 10.91(1.12) | 11.08(1.47) |  |  |  |
| Inattentiveness | 6.55 (.49) | 6.67 (.74) | .02  (1,21) | .89 | .001 |
| Hyperactivity-Impulsivity | 4.36 (.85) | 4.42 (.83) | .002  (1,21) | .97 | .0001 |

M: Male; Note that all the children were unmedicated.

**Supplementary Table S2.** Spontaneously Reported or Observed Adverse Events during transcranial random noise stimulation (tRNS) and sham stimulation, both combined with CT. The table indicates the number of participants (N) and the percentage of sessions endorsing side effects at some point during the intervention.

| Adverse event^#^ | tRNS + CT  (n = 11) | | Sham + CT  (n = 12) | |
| --- | --- | --- | --- | --- |
|  | N | % Sessions | N | % Sessions |
| Headache | 3 | 3 | 1 | 2 |
| Tingling | 3 | 4 | 1 | 1 |
| Itching | 9 | 27 | 10 | 33 |
| Local redness | 1 | 2 | 0 | 0 |
| Scalp burn | 1 | 1 | 1 | 1 |
| Scalp pain | 2 | 2 | 1 | 2 |
| Discomfort | 5 | 6 | 6 | 6 |
| Sleepiness | 0 | 0 | 0 | 0 |
| Mood change | 3 | 4 | 0 | 0 |
| Difficulty concentrating | 4 | 5 | 4 | 5 |
| Nausea | 0 | 0 | 0 | 0 |
| Dizziness | 0 | 0 | 0 | 0 |

**Supplementary Table S3.** Regression model of post-treatment secondary outcome scores, after covarying for baseline scores of each measure.

|  | β | | | Std Error | | | DF | | | t-value | | | p-value | |
| --- | --- | --- | --- | --- | --- | --- | --- | --- | --- | --- | --- | --- | --- | --- |
| Clinical symptoms (CGI-S) | | | | | | | | | | | | | | |
| Intercept | -2.36 | | | .98 | | | 22 | | | -2.38 | | | **.03*** | |
| Baseline | .55 | | | .19 | | | 20 | | | 2.85 | | | **.01*** | |
| Treatment | -.16 | | | .22 | | | 20 | | | -.73 | | | .48 | |
| Time | -.51 | | | .13 | | | 22 | | | -3.99 | | | **.0006***** | |
|  | | | | | | | | | | | | | | |
| WM (backward digit span scores) | | | | | | | | | | | | | | |
|  | **β** | | | **Std Error** | | | **DF** | | | **t-value** | | | **p-value** | |
| Intercept | -2.56 | | | .61 | | | 22 | | | -4.23 | | | **.0003***** | |
| Baseline | .44 | | | .09 | | | 20 | | | 5.08 | | | .**0001***** | |
| Treatment | .02 | | | .25 | | | 20 | | | .08 | | | .94 | |
| Time | .16 | | | .2 | | | 22 | | | .79 | | | **.44** | |
|  |  | | |  | | |  | | |  | | |  | |
| STM (forward digit span scores) | | | | | | | | | | | | | | |
| Intercept | -2.44 | | | .67 | | | 22 | | | -3.65 | | | **.001**** | |
| Baseline | .38 | | | .1 | | | 20 | | | 3.82 | | | **.001**** | |
| Treatment | -.28 | | | .32 | | | 20 | | | -.87 | | | .39 | |
| Time | .22 | | | .16 | | | 22 | | | 1.4 | | | .17 | |
|  | | | | | | | | | | | | | | |
| Executive function (BRIEF-Parents) | | | | | | | | | | | | | | |
| Intercept | | -3.39 | | | 1.08 | | | 22 | | | -3.14 | | **.005*** | |
| Baseline | | .06 | | | .02 | | | 20 | | | 3.55 | | **.002**** | |
| Treatment | | -.27 | | | .32 | | | 20 | | | -.84 | | .41 | |
| Time | | -.13 | | | .12 | | | 22 | | | -1.08 | | .29 | |
|  | |  | | |  | | |  | | |  | |  | |
| Executive function (BRIEF-Teachers) | |  | | |  | | |  | | |  | |  | |
| Intercept | | -6.55 | | | 1.02 | | | 19 | | | -6.43 | | **.00001***** | |
| Baseline | | .1 | | | .01 | | | 19 | | | 6.97 | | **.00001***** | |
| Treatment | | .26 | | | .23 | | | 19 | | | 1.09 | | .29 | |
| Time | | -.14 | | | .11 | | | 17 | | | -1.27 | | .22 | |
|  | | | | | | | | | | | | | | |
| Processing Speed (MOXO-CPT) | | | | | | | | | | | | | | |
| Intercept | | -3.84 | | | .63 | | | 22 | | | -6.12 | | .**00001***** | |
| Baseline | | .02 | | | .003 | | | 20 | | | 6.98 | | **.00001***** | |
| Treatment | | .02 | | | .23 | | | 20 | | | .08 | | .94 | |
| Time | | -.02 | | | .18 | | | 22 | | | -.1 | | .92 | |
|  | |  | | |  | | |  | | |  | |  | |
| Periodic RS-EEG activity: | | |  | | |  | | |  | | |  | |  |
| Beta power | | | | | | | | | | | | | | |
| Intercept | | | .04 | | | .2 | | | 56 | | | .22 | | .83 |
| Baseline | | | .66 | | | .07 | | | 56 | | | 9.8 | | **.0001***** |
| Treatment | | | -.09 | | | .16 | | | 56 | | | -.62 | | .54 |
| Time | | | .005 | | | .11 | | | 56 | | | .05 | | .95 |
| Beta center of frequency | | |  | | |  | | |  | | |  | |  |
| Intercept | | | -.53 | | | .6 | | | 56 | | | -.89 | | .38 |
| Baseline | | | .14 | | | .1 | | | 56 | | | 1.31 | | .2 |
| Treatment | | | .08 | | | .22 | | | 56 | | | .37 | | .72 |
| Time | | | -.11 | | | .2 | | | 56 | | | -.55 | | .58 |

Std= standard; DF= degrees of freedom; **p<0.005; ***p<0.0005

**Supplementary Table S4**. Blinding Integrity

| Intervention | Parent’s guess | | | | Question could not be asked | Active stimulation guess rate |
| --- | --- | --- | --- | --- | --- | --- |
|  | A | B | C | Total |  |  |
| tRNS + CT | 4 | 3 | 4 | 11 |  | 57% |
| Sham + CT | 7 | 2 | 1 | 10 | 2 | 78% |

A: believe treatment is active tRNS; B: believe the treatment is sham tRNS; C: unsure.

Note: Active stimulation guess rate (Fassi & Cohen Kadosh, 2020) was calculated as the rate of parents thought their children were received active treatment in each group (while excluding ‘unsure’ guesses).

**Supplementary Table S5**. A regression model of primary outcome measure post-treatment (t1) and at a 3-week follow-up (t2), as predicted from subjective intervention and not objective intervention (active/ sham)

|  | **Β** | | **Std Error** | **DF** | **t value** | **P value** |
| --- | --- | --- | --- | --- | --- | --- |
| **Clinical symptoms (ADHD-RS)** | | | | | | |
| Intercept | 2.69 | 4.25 | | 15 | .63 | .53 |
| Baseline score | .61 | .28 | | 13 | 2.15 | .05 |
| Subjective intervention^*^ | .69 | 2.37 | | 13 | .3 | .77 |
| Time | -.88 | .87 | | 15 | -1.01 | .33 |

^*^Subjective intervention—the participants’ subjective beliefs about receiving or not receiving an intervention
